# Preventing outbreaks in burn units: the role of hygiene compliance and early warning systems

**DOI:** 10.1186/s13756-025-01592-9

**Published:** 2025-07-01

**Authors:** Marie Lindblad, Fredrik Huss, Eva Tano, Birgitta Lytsy, Susanne Sütterlin

**Affiliations:** 1 Department of Surgical Sciences, Plastic Surgery, Uppsala University, Uppsala, Sweden; 2 Burn Centre, Department of Plastic and Maxillofacial Surgery, Uppsala University Hospital, Uppsala, Sweden; 3 Department of Medical Sciences, Section of Clinical Microbiology, Uppsala University, Uppsala, Sweden; 4 Department of Clinical Microbiology, Uppsala University Hospital, Uppsala, Sweden; 5 Department of Laboratory Medicine, Division of Clinical Microbiology, Karolinska Institute, Stockholm, Sweden; 6 Department of Women´S and Children´S Health, Paediatric Inflammation, Metabolism and Child Health Research International Maternal and Child Health, Uppsala University, Uppsala, Sweden; 7 Department of Paediatric Emergency and Infectious Diseases, Children’s Hospital, Uppsala University Hospital, Uppsala, Sweden

**Keywords:** Outbreak control, Hygiene errors, *Acinetobacter baumannii*

## Abstract

**Background and objectives:**

To analyse observations of staff’s hand hygiene, usage of gloves and plastic aprons, and dress code compliance at the Burn Centre, Uppsala University Hospital in Sweden, and to evaluate the association between hygiene non-compliance and outbreak periods. Furthermore, to explore the potential of using routine hygiene observations as an early warning tool for the risk of uncontrolled spread of (multidrug-resistant) bacteria.

**Methods:**

Direct observations of the personnel’s compliance with hand hygiene, usage of gloves and plastic aprons, and dress code were studied in relation to two *A. baumannii* outbreaks in 2014 and 2020. Interrupted time series analyses were conducted from 2013 to 2017 and 2018 to 2022 to evaluate outbreak intervention measures. Rolling sums of observed hygiene errors and 90th percentiles were calculated for four-, five-, and six-month periods.

**Results:**

During the study, 13,216 direct observations showed hygiene compliance ranging from 70 to 100% per category. Infection control interventions significantly reduced non-compliance after both outbreaks, with sustained improvements for two years following the first outbreak. Rolling four- to six-month sums, using 90th percentile thresholds of seven, nine, and eleven non-compliances predicted *A. baumannii* outbreaks.

**Conclusion:**

In this setting, compliance levels above 97% were consistently observed during outbreak-free periods, suggesting a potential protective effect. Focus on non-compliance as a key metric and rolling sums of non-compliance, may support early detection of increased outbreak risk and guide preventive interventions.

## Introduction

Outbreaks caused by multidrug-resistant bacteria, such as *Acinetobacter baumannii,* in intensive care units, are associated with high morbidity, mortality, and healthcare costs [1]. Transmission of such organisms can occur through various routes, including contact with healthcare personnel, contaminated surfaces or equipment, and environmental reservoirs [2, 3]. Infection Prevention and Control (IPC) measures such as hand hygiene, appropriate use of personal protective equipment, adherence to dress code, environmental cleaning, and staffing adequacy are all considered important factors in mitigating these risks.

Previous studies have demonstrated that improved hand hygiene compliance can reduce transmission of multidrug-resistant organisms, with increases from 50 to 70% being associated with outbreak containment in some settings [4, 5]. However, the specific compliance levels required to prevent outbreaks in high-risk environments such as burn intensive care units (ICUs) remain unclear [4, 6]. In practice, multiple IPC components interact, and the multifactorial nature of outbreaks poses challenges to identifying predictive indicators.

Routine structured observations of hygiene compliance are performed in many healthcare settings to monitor IPC performance and provide feedback to staff [7–11]. While these data are often used for quality improvement, their potential to serve as early warning signals for increased outbreak risk has not been fully explored.

In this study, we aimed to analyse eleven years of routine hygiene compliance observations at a burn ICU in Sweden. We focused on a composite measure of non-compliance encompassing hand hygiene, gloves and apron use, and dress code adherence. Our objective was to evaluate whether changes in non-compliance patterns were associated with two *A. baumannii* outbreaks and to explore whether these data could serve as an early warning system to support proactive IPC interventions.

## Methods

### Setting

The Burn Centre includes an intensive care unit with an operating room, an outpatient clinic, a hydrotherapy room, and a plastic surgery ward for minor burns. The Burn ICU admits approximately 120 patients and 80 minor burns annually to the plastic surgery ward. Minor burns are treated by the same plastic surgeons, physiotherapists, occupational therapists, and dietitians; however, they are cared for by the plastic surgery ward’s nurses. Wound care and surgery for the plastic ward burn patients are performed in the burn ICU’s outpatient clinic and operation room. The burn ICU comprises seven single rooms. Staffing in the burn ICU includes registered nurses, intensive care nurses, anaesthesia nurses, assistant nurses, as well as a team of plastic surgeons, and intensivists from the Department of Anaesthesia and Intensive Care.

### Systematic direct observations of compliance with hand hygiene, usage of gloves and plastic aprons, and dress code

Compliance with hand hygiene, usage of gloves and plastic aprons, and adherence to dress code was monitored through systematic, unannounced direct observations conducted by trained staff members. Seven key hygiene moments were continuously monitored. In the hand hygiene category, hands and forearms must be disinfected (Liv DES + 45 Isopropanol IPA 60, Clemondo AB, Helsingborg, Sweden) before (1) and after (2) patient contact. In the personal protective devices category, gloves should be worn when handling bodily fluids and surface disinfectants (3), and a disposable apron should be worn during patient care, contact with patient beds, and when handling contaminated items (4). In the dress code category, scrubs with short sleeves are to be used (5). Hands and forearms must be free of rings, watches, and bracelets (6). Hair should be set or kept short (7). Observed scored compliance was placed in a binary scale (1 = compliant, 0 = non-compliant). Each month, at least ten staff members from various professions, including physicians, nurses, and paramedics, were monitored, and results were recorded in the hospital database. Compliance data from January 2013 to August 2024 was extracted for analysis. For analytical purposes, we used a composite measure of non-compliance across all seven hygiene-related criteria, assigning a score of 1 for each instance of non-compliance. This approach aimed to reflect overall IPC performance and to enhance sensitivity for trend detection in a high-compliance environment.

Hygiene observations were conducted covertly by a small group of trained and experienced staff, typically nurses and assistant nurses working at the Burn Centre. The observers used a standardized checklist based on the national guidelines for hygiene observation developed by the Swedish Association of Local Authorities and Regions (SKR).

Observers were trained by IPC staff prior to initiating observation. The observations were conducted as part of routine hospital surveillance, independent of the current study, and observers were not aware of the here presented study. Measurement training for observing compliance with hand hygiene and dress code is conducted twice a year, with repeated meetings for hygiene observers. While formal inter-rater reliability was not assessed, consistency was supported by the limited number of observers, shared training background, and use of a structured protocol throughout the 11-year study period.

### Outbreak descriptions during study period

Between November 2014 and April 2015, the BICU suffered from an outbreak caused by an ST15 *A. baumannii*. The isolate was carbapenem-resistant due to OXA-23 production and affected nine patients, two of whom had a fatal outcome [7]. From February to July 2020, another outbreak occurred caused by an ST2 *A. baumannii*. The isolate was carbapenem-resistant due to OXA-23 and affected seven patients, none of whom had a fatal outcome. The outbreak was considered to have started when a patient treated at the Burn Centre was identified with a culture-positive sample for the outbreak strain, confirmed by pulsed-field gel electrophoresis (PFGE), arbitrarily primed polymerase chain reaction (AP-PCR), and subsequently by whole genome sequencing. The outbreak period was defined from the first to the last confirmed case, with continued active surveillance beyond this period to ensure containment [7].

Interventions to control the *A. baumannii* outbreaks consisted of prevention control targeting hand hygiene, usage of gloves and plastic aprons, dress code, and decontamination of environmental sources, such as e.g. surfaces and sinks. These interventions included, among other things, continuous education on hygiene practices, infection surveillance, cleaning routines, and the introduction of patient screening for *A. baumannii* colonization. All infection control interventions are described in detail elsewhere [7].

### Statistical calculations

#### Measures

High compliance rates lead to compressed ranges and reduced sensitivity for detecting subtle but clinically relevant changes over time. Therefore, non-compliance counts were chosen instead of compliance rates to enhance the detection of deviations from expected performance and to directly reflect potential IPC lapses. The study's primary dependent measure was monthly non-compliance with hygiene routines. The lowest possible number of instances observed each month was 0, and the largest possible number of instances observed each month was 70. Although a higher number of observations occurred during some months, all data were adjusted to reflect the equivalent of 10 observations per hygiene category per month and 70 in total.

#### Interrupted time series

An interrupted time series analysis was used to estimate the interventions'effects on non-compliance with hygiene routines. The analysis included two intervention periods corresponding to infection prevention control interventions implemented during the *A. baumannii* outbreaks in 2014 and 2020. The first intervention period spanned from January to April 2015, and the second from May to July 2020. The interrupted time series analyses aimed to evaluate whether the interventions significantly reduced counts of non-compliance with hand hygiene, usage of gloves and plastic aprons, and dress code practices. To minimize the likelihood of introducing confounding factors and reduce the effects of unrelated variations in data over time, all pre- and post-intervention periods were standardized to 24 months. The first period covered January 2013 to April 2017, while the second period spanned May 2018 to July 2022.

The interrupted time series models included two independent variables, *Timeperiod* and *Interruption at outbreak*, along with an interaction term, to capture the effect of the intervention over time (Time period*Interruption at outbreak). Data pre-processing included standardizing measurement frequencies across periods, as some periods had more frequent measurements. These were adjusted to a common denominator to ensure comparability across time points. The model is specified as follows:

$$Observed\;non-compliances\:=\:intercept\:+\;\beta_1\ast Timeperiod\:+\;\beta_2\ast Interruption\;at\;outbreak\:+\;\beta_3\ast Timeperiod\ast Interruption\;at\;outbreak.$$

The observed non-compliance with hygiene routines constituted count data, which were expected to follow a Poisson distribution [12]. The regression models for the time series analysis were estimated using log links, and graph curves were plotted by exponentiations of the sums of the regression model terms. To assess potential problems with autocorrelation in the non-compliance measure, Durbin-Watson statistics were computed using the *car* package in R [13]. Durbin-Watson statistics between 1.5 and 2.5 are conventionally considered acceptable, as they suggest that autocorrelation is small or very small [14].

#### Rolling sums and thresholds

In order to assess the risk and timing of outbreak occurrences, rolling sums of instances of non-compliance with hand hygiene, usage of gloves and plastic aprons, and dress code (x) were calculated for four-, five-, and six-month periods (w). For rolling sums at time point t, the value *RS*_*t*_ is calculated as the sum of values x_i_ over a rolling period of size w.

$${RS}_t=\sum\limits_{i=t-w+1}^t\;x_i$$

Alert thresholds *T* were calculated as 90th percentile (*P*_90_) of rolling sums (*RS*_*t*_), using an interpolation-based method. These thresholds were determined separately for each rolling period and for the entire observation period from January 2013 to August 2024.

$$T={P}_{90}{(RS}_{t})$$

Kaplan–Meier survival analysis was conducted to estimate the probability of an outbreak occurring over time after predefined non-compliance thresholds (90th percentiles), with 95% confidence intervals. Log-rank tests were used to statistically compare survival curves between groups, assessing whether different rolling periods and thresholds significantly affected time to outbreak. All analyses were performed using the R packages *survival* [15] and *zoo* [16].

This study was based on anonymized routine quality improvement data collected as part of ongoing IPC monitoring. No personal identifiers were included, and no patient-specific data were analyzed. Given the Declaration of Helsinki, a request was made to the Swedish Ethical Authority and the project was deemed exempt from ethical review as per Swedish legislation on quality improvement initiatives. Human Ethics and Consent to Participate declarations: not applicable.

## Results

### Observed non-compliances with hand hygiene, usage of gloves and plastic aprons, and dress code

Between January 2013 and August 2024, 13,216 observation measurements were recorded in the hospital’s database for the burn ICU. Overall compliance with hand hygiene, usage of gloves and plastic aprons, and dress code ranged between 70 and 100% per month and category, corresponding to cumulative non-compliance ranging from 0% to 5.9% across all three categories. Non-compliance was assessed as a composite measure of seven hygiene-related practices, with each non-compliant observation contributing one unit to the total score.

The highest cumulative non-compliance rates were observed within the hand hygiene category, particularly before the first intervention, with a 5.9% non-compliance rate (27/460), corresponding to a median compliance per month of 95% (75%—100%). Non-compliance in using gloves and plastic aprons was also the highest in this period at 2.4% (11/460), while nearly no mistakes were noted in dress code compliance. There were missing observations for May, August, September 2023 and July 2024 (Table 1).

**Table 1:** Observation results of compliance and non-compliance to hand hygiene and dress code practices before and after infection control interventions during *A. baumannii* outbreaks that occurred at the Burn Centre, Uppsala University Hospital, Sweden, during 2014 and 2020, respectively. Total non-compliances are given as per total direct observations and the rate of non-compliances were calculated for each period and categories of hand and hygiene, usage of gloves and plastic aprons and dress code. Compliances rates per month are given as median with maximum and minimum range for each period and categories

	***A. baumannii* outbreak 2014**		***A. baumannii* outbreak 2020**	
	**Before intervention**	**After intervention**	**Before intervention**	**After intervention**
	**January 2013 to December 2014**	**May 2015 to December 2017**	**January 2018 to April 2020**	**August 2020 to August 2024**
**Hand Hygiene**				
Cumulative non-compliance per period	27/460 (5.6%)	7/620 (1.1%)	10/562 (1.8%)	35/1,662 (2.2%)
Compliance per month during respective period	95% (75%—100%)	100% (90%—100%)	100% (85%—100%)	100% (70%—100%)
**Plastic protective devices**				
Cumulative Non-compliance per period	11/460 (2.4%)	6/620 (0.5%)	6/562 (1.1%)	24/1,662 (1.5%)
Compliance per month during respective period	100% (80%—100%)	100% (90%—100%)	100% (90%—100%)	100% (70%—100%)
**Dress code**				
	1/690 (0.1%)	0/930 (0%)	0/1,124 (0.0%)	16/3,284 (0.5%)
	100% (97%—100%)	100% (100%—100%)	100% (100%—100%)	100% (95%—100%)
**All categories**				
Cumulative Non-compliance per period	39/1,610 (2.4%)	10/2,170 (0.4%)	16/2,248 (0.7%)	75/6,528 (1.1%)
Compliance per month during respective period	98% (84%—100%)	100% (93%—100%)	100% (92%—100%)	100% (78%—100%)

### Interrupted time series analysis

Due to high compliance rates with hand hygiene, the usage of gloves and plastic aprons, and dress code, calculations on interrupted time series, and rolling sums, were performed on non-compliance. A Poisson regression model [12] was employed to analyse trends in non-compliance with hand hygiene, the usage of gloves and plastic aprons, and dress code before and after the interventions implemented from January to April 2014 and May to July 2020. For the first period, the pre-intervention trend for non-compliance over time was positive but not statistically significant (*r* = 0.03, *p* = 0.14). The model indicated no significant change in non-compliance level immediately following the intervention (*r* = 1.57, *p* = 0.25). However, non-compliance significantly declined over time, as indicated by a negative interaction term (*r* = −0.1054, *p* = 0.016). This suggests that the intervention had a sustained, positive effect over time, gradually reducing non-compliance. Autocorrelation was assessed using the Durbin-Watson test [13], yielding a value of 1.67, indicating no significant autocorrelation in the residuals. The post-intervention analysis revealed a statistical significance (*r* = −0.040, *p* < 0.001), indicating a consistent reduction in non-compliance over time after the intervention.

**Fig. 1:**
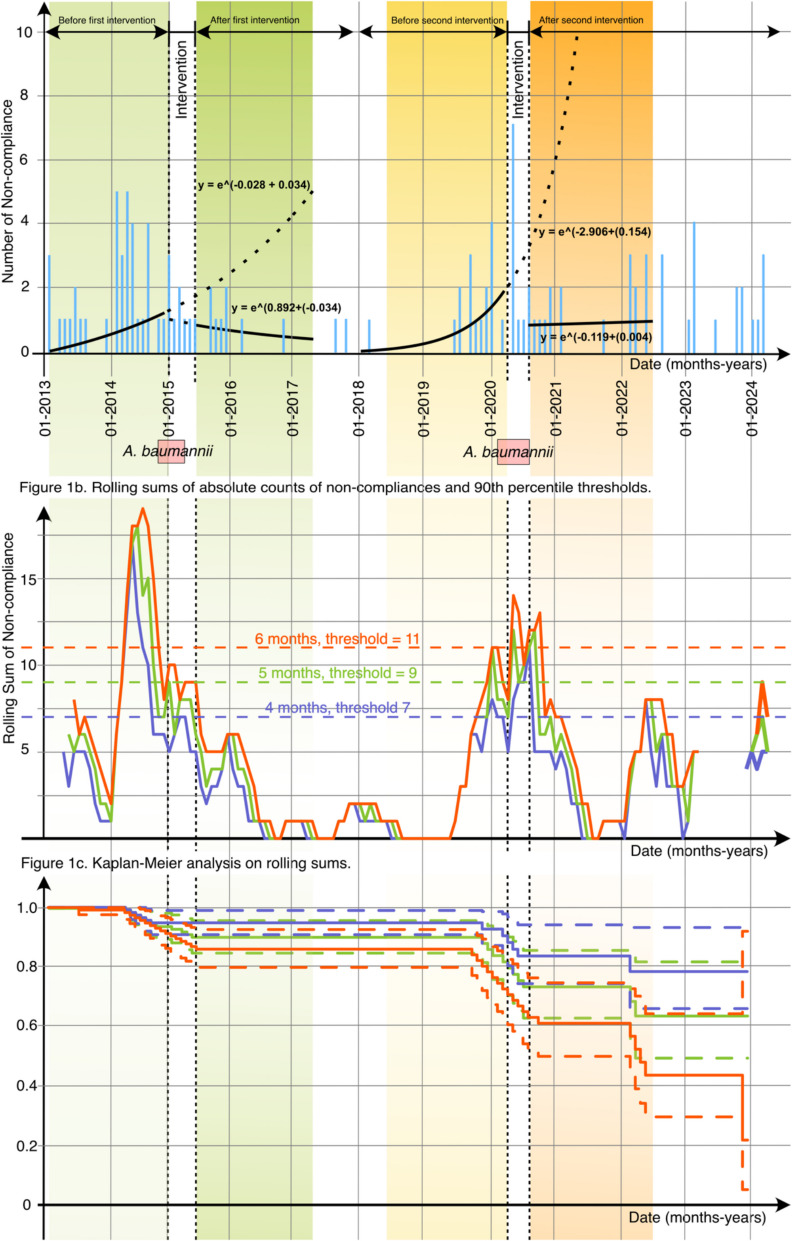
**a** Graphical illustration of observed non-compliances to hand hygiene, usage of gloves and plastic aprons and dress code practices (blue staples) over time, with the intervention period marked by dashed vertical lines. The solid line before the intervention represents the pre-intervention trend, and dotted lines during and following intervention show the counterfactual trends, reflecting a modelled change in non-compliance over time. The immediate level change at the time of intervention and the subsequent change in trend post-intervention are illustrated, capturing the immediate and long-term effects of the intervention on hygiene practices. Above the graph, the periods of the two *A. baumannii* outbreaks are marked. **b** Rolling sums of observed non-compliances were calculated for four-month (purple), five-month (green), and six-month (red) periods. Thresholds based on the 90th percentiles of these rolling sums are indicated by dotted horizontal lines: for four-month periods with a threshold of 7 (purple), five-month periods with a threshold of 9 (green), and six-month periods with a threshold of 11 (red). **c** Kaplan–Meier analysis was performed on rolling sums for combinations of four-month (purple), five-month (green), and six-month (red) periods, each with respective thresholds and associated 95% confidence intervals

For the second period, from May 2018 to July 2022, the pre-intervention trend for non-compliance over time was positive and statistically significant (*r* = 0.153, *p* = 0.002), indicating an increase in non-compliance before the intervention. The model also showed an immediate change in non-compliance following the intervention (*r* = 2.788, *p* = 0.078)), suggesting an initial effect at the time of implementation of infection control interventions. A negative interaction term suggested that non-compliances decreased over time (Interaction_term_Timeperiod*Interruption_at_outbreak (β3) = 2.789, *p* = 0.008). During the post-intervention period of the second outbreak, no significant trends in non-compliance over time were observed (*Timeperiod* (β1) = −0.005, *p* = 0.892). This suggests that while the intervention maintained a stable level of compliance, it did not lead to further reductions in non-compliance during the post-intervention period. Assessment of autocorrelation using the Durbin-Watson test [14] yielded a value of 1.89, indicating no significant autocorrelation in the residuals (Fig. 1a).

### Rolling sums and thresholds

Rolling sums of absolute non-compliance counts per month were calculated over four-, five-, and six-month periods, resulting in 90th percentile alert thresholds of seven, nine-, and eleven rolling summed non-compliances, respectively. Each rolling sum period with its corresponding threshold preceded an outbreak period, suggesting a potential association with increased outbreak risk. The earliest alerts occurring eight months before the first outbreak, five months before subsequent outbreaks for four months, and four months before five- and six-month periods.

The survival curves indicated a progressively decreasing survival probability across all rolling periods prior to *A. baumannii* outbreaks. Although log-rank testing did not reveal statistically significant differences between threshold levels or rolling periods (Chi-squared = 3.4, df = 8, *p* = 0.9), survival curves consistently showed decreasing probability of remaining outbreak-free following threshold exceedance (Fig. 1b and c).

Based on the 90th percentile thresholds identified in the rolling sum analyses, the theoretical maximum allowable non-compliance per month before outbreak risk increases is approximately 1.8 across all observed hygiene categories. This equates to a theoretical rate of 1 to 2 non-compliances per 70 observations (ten observations across seven categories per month), corresponding to a 97% compliance threshold at which the risk for the *A. baumannii* outbreak begins to increase.

## Discussion

Outbreaks of multidrug-resistant organisms, such as *Acinetobacter baumannii*, are typically multifactorial in nature, and environmental contamination, water quality, equipment sterilization, and staffing levels have all been implicated in previous outbreaks. Within this broader context of transmission dynamics, our study concentrated on a specific, measurable, and routinely collected aspect of IPC performance—compliance with hygiene protocols—as a potential indicator of outbreak risk.

Over the eleven-year observation period (2013–2024), median compliance with hand hygiene, glove and apron use, and dress code in the Burn Intensive Care Unit (BICU) at Uppsala University Hospital remained high, ranging from 98 to 100%. The highest rate of cumulative non-compliance to hygiene protocols was observed before the first *A. baumannii* outbreak in 2014, with a 5.9% non-compliance rate in hand hygiene. Infection control interventions reduced non-compliance after both outbreaks, with a statistically significant immediate decrease following the second outbreak—the long-term trend after the first outbreak showed a sustained, significant reduction in non-compliance for two years. In contrast, non-compliance trends increased slightly over the two years following the second outbreak.

The World Health Organization [17] and others [4, 18] recommend hand hygiene compliance monitoring, which is widely implemented in Swedish hospitals. While 100% compliance with hygiene protocols is ideal, compliance levels often fall significantly short and vary widely globally and within Swedish hospitals [18–20]. Although overall compliance at the burn ICU was high, two outbreaks of multidrug-resistant *A. baumannii* still occurred. The 90th percentile thresholds from rolling sums corresponded to 1–2 observed monthly non-compliances across all seven categories, based on standardized observation volumes. The actual number of non-compliance events, including those not captured through observation, was likely higher. This level corresponded to an estimated compliance rate of approximately 97%, which in our setting was only observed during outbreak-free periods. The analyses in this study focused on non-compliance rates rather than the compliance rates more commonly reported in the literature [21–23].

Focusing on non-compliance instead of compliance has statistical advantages in high-adherence settings due to the ceiling effect for compliance above 95%. By analysing the number of non-compliance directly, it becomes easier to detect subtle but clinically relevant changes and can also serve as a proxy for increased transmission risk. While this does not establish causality, it offers a practical way to identify early signs of declining overall IPC performance. To be effective, however, such metrics must be communicated thoughtfully. Feedback focused solely on non-compliance can negatively affect morale, especially if perceived as punitive [24]. Interestingly, negative feedback does not always diminish motivation. A neuroscience study found that receiving negative feedback in one task can enhance intrinsic motivation in a subsequent competence-supportive task, likely due to a restorative drive to regain competence [25]. This suggests that when framed constructively, supportively and with awareness of the individual [26], non-compliance feedback may help reinforce engagement rather than reduce it.

Infection control interventions implemented during the *A. baumannii* outbreaks significantly reduced hygiene errors and contributed to the successful containment of the first outbreak [7]. The second outbreak coincided with the SARS-CoV-2 pandemic, during which compliance targets for hygiene practices were raised to 100%. However, a shortage of specialized nurses and the suspension of elective surgeries in the plastic surgery department led to a situation where nursing staff were reassigned to SARS-CoV-2 wards. Simultaneously, plastic surgeons, were assigned to participate in nursing patients. These factors significantly contributed to the reduced effectiveness of the infection control interventions during the second outbreak. Additionally, a change in observers led to more rigorous direct observations with less tolerance for compliance failures. During periods, the unit went through a high staff turnover, which may explain the gradual increase in non-compliance with hygiene practices.

Aggregated time-series measures are well established in public health and health care, often used as early warning systems for outbreak situations [27, 28]. When utilizing a 90th percentile threshold, the rolling sums method is straightforward to implement in healthcare settings already performing hygiene observations. Its primary advantage lies in its ability to signal increased risks before an outbreak occurs, providing an opportunity for preventive action rather than reactive measures after the outbreak. Shorter periods for calculating rolling sums may be more susceptible to variation and could trigger warnings earlier than longer periods. Furthermore, variability in observation frequency across periods could affect the precision of threshold estimates, and therefore, the 97% compliance threshold identified in this dataset may not generalize directly to other settings or institutions, where differences in observation practices or infection dynamics could yield different results [29]. In the context of antimicrobial resistance, the ability to detect early signals of lapses in IPC performance is critical. Proactive identification of hygiene non-compliance may offer a valuable adjunct to molecular surveillance in preventing clonal spread of MDR pathogens, especially in high-risk settings such as burn ICUs.

The strength of this study lies in the low level of missing data, which was limited to four months during the final two years of the observation period, providing a robust dataset spanning over eleven years. Additionally, the study includes two well-documented *A. baumannii* outbreaks. The retrospective nature of the data analysis is a limitation, and validating the rolling sums and associated thresholds in a prospective controlled study would be preferable. Nevertheless, evaluating outbreak prevention poses challenges, as implementing an intervention and control group would not be ethically feasible. While the high levels of hand hygiene and dress code compliance observed in this study may appear unusually elevated, the authors believe they reflect the specific institutional context at Uppsala University Hospital and the Burn Centre ICU. Hygiene and infection prevention have long been prioritized at both leadership and unit level, supported by systematic monitoring and an active safety culture. Staff receive regular reminders, short refresher trainings, and engage in peer-to-peer feedback around hygiene practices.

## Conclusion

In summary, this study highlights that in this high-risk setting, outbreak risk appeared to increase when compliance levels fell below approximately 97%. While this may serve a useful internal benchmark, further studies are needed to validate such thresholds in other contexts. Focusing on non-compliance as a key metric, and applying rolling sums over time, may support early detection of deteriorating IPC performance and guide timely preventive action. The method’s simplicity and compatibility with existing monitoring system make it a promising tool for infection control and offers a practical and scalable complement to traditional surveillance strategies in the ongoing effort to curb the spread of antimicrobial-resistant pathogens.

## Data Availability

The datasets used and analysed during the current study are available from the corresponding author on reasonable request.
